# Branching Off: New Insight Into Lysosomes as Tubular Organelles

**DOI:** 10.3389/fcell.2022.863922

**Published:** 2022-05-11

**Authors:** K. Adam Bohnert, Alyssa E. Johnson

**Affiliations:** Department of Biological Sciences, Louisiana State University, Baton Rouge, LA, United States

**Keywords:** aging, autophagy, cell biology, lysosome morphology, organelles, tubular lysosomes

## Abstract

Lysosomes are acidic, membrane-bound organelles that play essential roles in cellular quality control, metabolism, and signaling. The lysosomes of a cell are commonly depicted as vesicular organelles. Yet, lysosomes in fact show a high degree of ultrastructural heterogeneity. In some biological contexts, lysosome membranes naturally transform into tubular, non-vesicular morphologies. Though the purpose and regulation of tubular lysosomes has been historically understudied, emerging evidence suggests that tubular lysosomes may carry out unique activities, both degradative and non-degradative, that are critical to cell behavior, function, and viability. Here, we discuss recent advances in understanding the biological significance of tubular lysosomes in cellular physiology, and we highlight a growing number of examples that indicate the centrality of this special class of lysosomes to health and disease.

## Introduction

As the major digestive organelle in the cell, the lysosome is uniquely equipped to recalibrate cellular homeostasis on demand. Lysosomes act as central hubs for several cellular-degradation pathways. During the process of autophagy, for example, lysosomes receive cytosolic cargo *via* autophagosome delivery or direct uptake, and once cargo is within the lysosomal compartment, acid hydrolases activated by low pH digest the material (Kaur and Debnath 2015; Ballabio and Bonifacino 2020; Butsch et al., 2021). In addition to destroying material originating from within the cell *via* autophagy, lysosomes receive and degrade material delivered from outside the cell *via* endocytosis and phagocytosis (Luzio et al., 2009; Nguyen and Yates 2021). Beyond these digestive tasks, lysosomes also serve many non-degradative roles in the cell, including in nutrient sensing, intracellular sorting, metabolism, and signaling (Settembre et al., 2013; Lim and Zoncu 2016; Perera and Zoncu 2016; Trivedi et al., 2020; Bouhamdani et al., 2021). Given these important activities, lysosome dysfunction is often catastrophic for a cell and can lead to aging and degenerative pathologies (Futerman and van Meer 2004; Rubinsztein 2006; Settembre et al., 2008). Understanding how lysosomes operate to support cellular and organismal health has emerged as an important current topic in biomedicine, given their broad implications for disease prevention and treatment.

**FIGURE 1:**
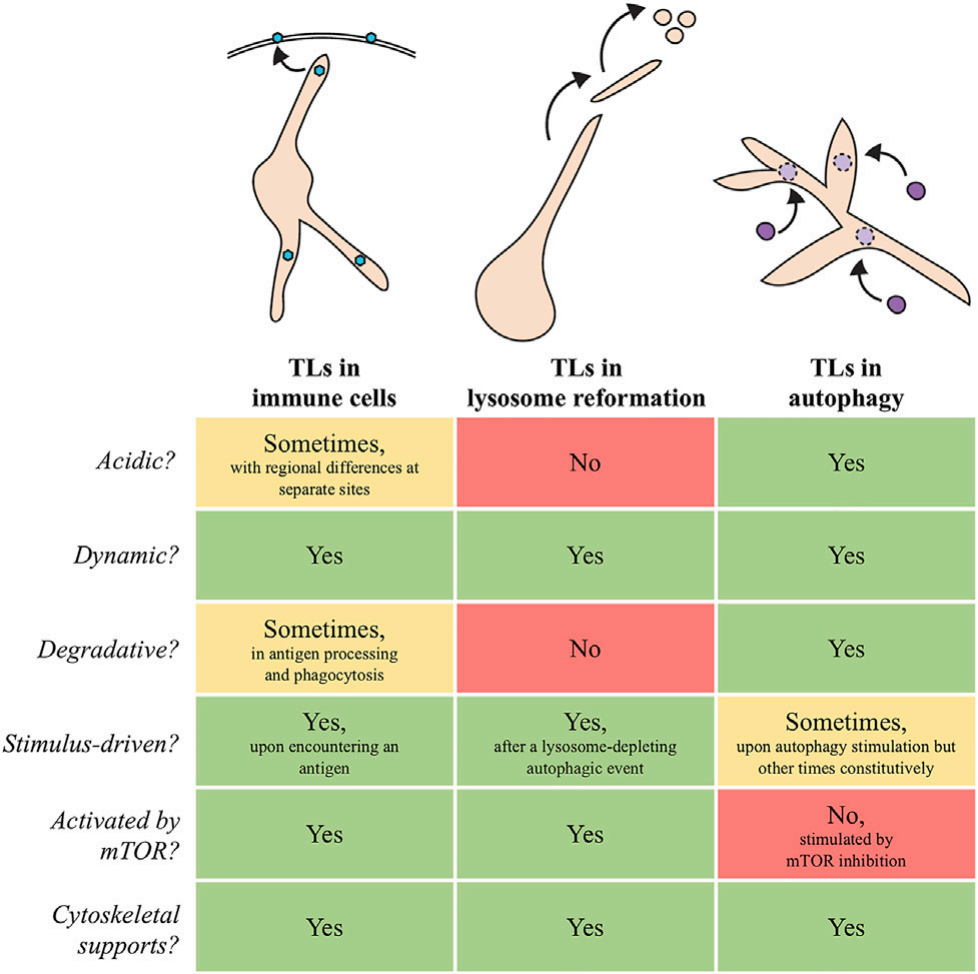
Schematic models and summary of similarities and differences between TLs in immune cells, lysosome reformation, and autophagy. Blue hexagons in immune cells: antigens. Purple circles that dock and fuse with autophagic TLs: autophagosomes.

Despite their importance to animal health and disease, the common perception of lysosome structure and function is quite simplistic; lysosomes are traditionally depicted as discrete, vesicular organelles, though this is not always the case. It has been known for several decades that lysosome membranes are capable of forming extended tubular projections (Phaire-Washington et al., 1980; Swanson et al., 1987a; Knapp and Swanson 1990; Saftig and Klumperman 2009; Johnson et al., 2016), suggesting heterogeneity in lysosome morphology. Moreover, the lysosomes in a cell can vary from each other by additional measures, including size, location, pH, and enzyme composition (Heuser 1989; Allen et al., 2006; Bandyopadhyay et al., 2014; Johnson et al., 2016; Li et al., 2016; Cheng et al., 2018). Interestingly, maintenance of these differences may be disrupted in disease states (Gowrishankar et al., 2015; Johnson et al., 2021), underscoring the importance of understanding the unique qualities that define different types of lysosomes. In this mini-review, we highlight three major examples of tubular lysosomes (TLs) that form in various biological contexts (Figure 1), and we discuss their implications for cellular health and homeostasis.

## TLs in Immune Responses

Lysosome tubulation was first documented in 1980 in bone-derived cultured macrophages (Phaire-Washington et al., 1980). As macrophages are phagocytic immune cells, their primary function is to engulf and degrade foreign pathogens and deliver the contents to the lysosome for degradation (Levin et al., 2016). When macrophages are in a resting, unstimulated state, lysosomes show a peri-nuclear localization and a vesicular morphology; however, upon stimulation with an agonist, lysosomes transform into extended tubules that radiate from the cell center and, in some cases, form an interconnected web-like network throughout the cytoplasm (Swanson et al., 1987a; Knapp and Swanson 1990). Although the exact function of TLs in activated macrophages is still unclear, TL network expansion increases the internal fluid volume and surface area of lysosomes (Swanson et al., 1987b; Knapp and Swanson 1990; Hipolito et al., 2019). Notably, lysosomal protein levels scale with the expanded endo-lysosomal system by a mechanism that is independent of TFEB (Hipolito et al., 2019), the major transcription factor that regulates lysosome biogenesis (Settembre et al., 2013). Instead, increased lysosomal protein levels in this context are dependent on mTOR-dependent translational regulation (Hipolito et al., 2019). Thus, it has been proposed that the increased surface area and volume of TL networks may accelerate turnover of phagocytosed material by providing more docking sites for vesicle fusion and increasing the internal holding capacity of lysosomes.

Dendritic cells (DCs) are a second major type of immune cell in which TLs have been described. Dendritic cells continually survey the environment for foreign antigens, and, when they encounter an antigen, they internalize it *via* the endocytic system and process it for presentation to CD4^+^ T-cells. During intracellular processing, antigens assemble with MHC Class II molecules in late endosomes and lysosomes (Savina and Amigorena 2007). In this context, lysosomes execute a uniquely specialized non-degradative role. In fact, lysosomes in DCs are often referred to as MHC-II compartments, as their main role is to house MHC-II molecules and internalized antigens in anticipation of an immune response. Upon immune stimulation, lysosomes in DCs also act as mobilization vehicles; in response to a stimulus, the lysosomal compartments tubulate to the cell periphery and aid in the presentation of antigen-loaded MHC-II molecules on the cell surface (Barois et al., 2002; Chow et al., 2002). Despite their unique functions, these atypical lysosomes still harbor canonical lysosomal proteins, such as LAMP-1, and they stain with LysoTracker, a dye that marks acidic compartments (Vyas et al., 2007). How some proteins within these TLs, including the antigen themselves, are selectively rescued from degradation is still unknown, but LysoTracker staining within DC tubular networks is non-uniform (Vyas et al., 2007), suggesting that local microenvironments with different molecular properties might exist at separate locations within these TLs.

Mechanistically, TLs in immune cells require support of the microtubule cytoskeleton and motor proteins (Kinesin-1 and Dynein) for their extension and movement to the cell periphery (Hollenbeck and Swanson 1990; Vyas et al., 2007; Mrakovic et al., 2012). TL dynamics are further promoted by the activity of two GTPases, Rab7 and Arl8B, that along with multiple co-factors link TLs to either Kinesin-1 or Dynein to promote bidirectional movement along microtubules (Jordens et al., 2001; Vyas et al., 2007; Pankiv et al., 2010; Mrakovic et al., 2012). Although TLs can move bidirectionally, tracking TL movement with the plus-end microtubule binding protein EB1 indicates that TLs predominantly move towards the plus-end of microtubules (Vyas et al., 2007), which is consistent with their outward radial movements to the immune-cell periphery. It has been proposed that Dynein acts as an anchor to stabilize TLs on microtubules, while kinesins play a more active role in their extension (Hipolito et al., 2018). This activity is likely coupled to mechanisms that supply additional lysosomal membrane material to promote the long tubule extensions observed; however, the molecular nature of these mechanisms has yet to be described.

While it is clear that TL induction is tightly coupled to immune-cell activation, the signaling pathways that relay information to trigger lysosome remodeling into tubular networks when immunogenic antigens are detected is not yet entirely clear. While phagosome formation itself is not a prerequisite for TL formation (Vyas et al., 2007), it has been found that TL induction requires the activity of MyD88-dependent phagosomal Toll-like Receptor (TLR) signaling in DCs (Boes et al., 2003; Mantegazza et al., 2014) and mTOR activity in macrophages (Saric et al., 2016). In DCs, activation of TLR signaling is accompanied by increased expression of two H^+^/oligopeptide symporters, SLC15A3 and SLC15A4, that reside at the lysosome membrane and export di- and tri-peptides from the lysosomal lumen to the cytosol (Nakamura et al., 2014). Disruption of either transporter impairs lysosome tubulation upon stimulation, whereas overexpression of either transporter alone is sufficient to induce lysosome-membrane tubulation (Nakamura et al., 2014). Overexpression of other endo-lysosomal transporters has not been found to induce lysosomal tubulation (Nakamura et al., 2014); thus, the specific increase in SLC15A3 and SLC15A4 at lysosomal membranes might directly aid in membrane expansion and tubulation in DCs.

## TLs in Lysosome Reformation

After a major autophagic event, the pool of functional lysosomes in a cell dramatically declines. To synthesize new lysosomes *de novo* would seem energetically demanding and time consuming; thus, the cell has devised a recycling mechanism to repopulate lysosomes in bulk. This mechanism, termed autophagic lysosome reformation (ALR), allows lysosomes to be regenerated from pre-existing autolysosomes (Yu et al., 2010). ALR requires mTOR reactivation and, thus, occurs only after autophagy has terminated (Yu et al., 2010). During ALR, proto-lysosome tubules emanate from autolysosomes. These tubules are LAMP1^+^, but they are devoid of autophagic cargo and hydrolytic enzymes (Yu et al., 2010). They also do not stain with LysoTracker, indicating that they are not acidic (Yu et al., 2010). Eventually, vesicles fission from the tubules and undergo a maturation process to re-acidify and become functional lysosomes.

Similar to TLs described in immune cells, ALR-specific autolysosome tubules show dynamic behaviors that depend on the microtubule cytoskeleton, and Kinesin-1 and Dynein motors (Yu et al., 2010; Du et al., 2016). Lysosomes are tethered to Dynein *via* the EF-hand-containing protein ALG-2, which in concert with the Ca^2+^ channel TRPML regulates lysosome tubulation during prolonged starvation (Li et al., 2016). There is also evidence that ALR relies heavily on changes to lipid composition at autolysosomal membranes. For example, selective enrichment of PI(4,5)P_2_ is needed for ALR initiation and proceeds *via* the action of multiple lipid kinases, including PIP5K and PI4K (Rong et al., 2012; Sridhar et al., 2013). Moreover, increased PI(3,5)P_2_ is necessary for TRPML-dependent lysosome tubulation (Li et al., 2016). In addition to these regulators, a few other factors have been identified to modulate ALR, including the putative sugar transporter Spinster (Spin), the vesicular-trafficking protein Clathrin, and the actin-nucleation promoting factor WHAMM (Rong et al., 2011, 2012; Dai et al., 2019). Accordingly, multiple branches of regulation appear to converge to ensure efficient lysosome tubulation during ALR.

In a parallel mechanism, lysosomes can also be reformed from phagosomes, rather than autolysosomes (Levin-Konigsberg et al., 2019; Lancaster et al., 2021). Like ALR, phagosomal lysosome reformation (PLR) is only triggered when the pool of functional lysosomes is severely depleted by multiple rounds of phagocytosis (Lancaster et al., 2021). Moreover, tubulation events during PLR are also dependent on the microtubule cytoskeleton for dynamic support and Clathrin to promote membrane fission (Lancaster et al., 2021). Changes in phagosomal membrane lipid composition also seem to be critical during the process of phagosome resolution; phagosomes tether to the ER *via* the Rab7 effector ORP1L and transfer PtdIns(4)P to the ER membrane, generating alternating areas along the membrane that are either rich or depleted of PtdIns(4)P (Levin-Konigsberg et al., 2019). Tubulation initiates at sites of PtdIns(4)P-rich regions, where two small GTPases, ARL8b and SKIP, are selectively enriched to recruit Kinesin motor proteins (Levin-Konigsberg et al., 2019). It has also been proposed that physical linkage to the ER provides a central anchor to oppose the physical force of phagosomal membrane tubulation to the cell periphery (Levin-Konigsberg et al., 2019).

Given that the major function of lysosome reformation processes is to replenish depleted pools of functional lysosomes after major autophagic or phagocytic events, dysfunctional lysosome reformation would be predicted to lead to a decrease in the number of functional lysosomes with time. Indeed, defects in ALR have been connected to multiple lysosome storage diseases and degenerative diseases. For example, inhibition of either *spastizin*/*SPG15* or *spatacsin*/*SPG11*, the two major genes associated with hereditary spastic paraplegia (HSP), results in accumulation of autolysosomes and depletion of free lysosomes, indicating their critical role in ALR (Chang et al., 2014). SPG15 directly associates with PI3P in lysosomal membranes *via* its FYVE domain and is necessary to initiate lysosome tubulation (Chang et al., 2014). These observations were also verified in an *in vivo* mouse model of HSP, further strengthening a direct link between ALR dysfunction and HSP (Varga et al., 2015). ALR dysfunction has also been linked to Parkinson’s Disease (PD); mutations in Glucocerebrosidase/GBA1 increase the risk of PD, and GBA1 deficiency results in defective ALR (Magalhaes et al., 2016). Collectively, these studies underscore the physiological importance of this tubulation-dependent, organelle-recycling process.

## TLs in Autophagy

In cell biology, lysosomes are perhaps best known for their defining role in the degradation of cellular cargo; this includes the process of autophagy. Historically, the identification of TLs in immune-cell activation and ALR suggested that lysosome tubules may function most predominantly in non-autophagic responses (*see* above). However, emerging evidence now indicates that TLs also play key roles in autophagic degradation of cellular material, and that TLs likewise influence animal physiology and disease as part of this process.

As in ALR, the protein Spin marks extensive networks of TLs in *Drosophila* muscle, albeit in this case the TL networks are present constitutively. These TLs span the muscle sarcoplasm in high density but remain spatially distinct from other tubular organelles, such as the mitochondria and endoplasmic reticulum (Johnson et al., 2015). Individual tubules within these networks show dynamic cycles of growth and shrinkage, like non-autophagic TLs in immune cells and cells undergoing ALR, yet fly muscle TLs also show a distinguishing property: they fuse with autophagosomes (Johnson et al., 2015). Inhibiting TL formation leads to an increase in ubiquitinated cytosolic cargo, suggesting the tubular morphology is important to support autophagic activity in this system (Johnson et al., 2015). Valosin-containing protein (VCP), a AAA-ATPase that when dysfunctional incapacitates damage clearance and leads to severe degenerative pathologies (Watts et al., 2004; Weihl et al., 2009), is one factor required for autophagic TL biogenesis and maintenance in fly muscle (Johnson et al., 2015). Molecularly, SVIP (small-VCP interacting protein) recruits VCP to lysosomes to establish the tubular morphology (Johnson et al., 2021). Overexpressing *SVIP* increases TL network density, and is sufficient to promote enhanced survival and stress resistance, remarkably even in the presence of lysosome inhibitors (Johnson et al., 2021). Several VCP disease mutations block the VCP-SVIP interaction and also engender TL network collapse and autophagic dysfunction (Johnson et al., 2021; Wall et al., 2021), indicating that loss of this lysosome morphology may underlie some aspects of degenerative disease pathology, perhaps in association with defective autophagy.

Though autophagic TLs may execute constitutive, homeostasis-enhancing functions by default in some cell types, including muscle, their activation appears stimulus-driven in other scenarios. Remarkably, various stimuli associated with bulk degradation of material have been demonstrated to promote TL formation and activity in multiple species. In the nematode *Caenorhabditis elegans*, molting, a process that entails significant proteolytic turnover to shed the outer cuticle layer of the worm, induces TL formation in the epidermis (Miao et al., 2020). In this case, molting-driven separation of the extracellular matrix from epidermal cells stimulates TL assembly, which coincides with increased expression of key lysosome-activating genes, including the lysosome-acidifying V-ATPase (Miao et al., 2020). Notably, mutations that abrogate TL formation during molting are associated with defects in cuticle replacement (Miao et al., 2020), suggesting that TL activity supports this developmental remodeling. *Drosophila* metamorphosis presents an interesting corollary. During this process, larvae transform into adult flies, a transition that requires massive tissue degradation and remodeling in order to generate new adult tissues. As in molting, loss of TL induction during metamorphosis, including by *spin* mutation, jeopardizes tissue-remodeling efficiency (Murakawa et al., 2020). One reason proposed for why TLs may be particularly well-suited for developmental-remodeling events is that TL networks, with their large surface area, provide an expansive platform to accommodate the docking and fusion of more autophagosomes than traditional vesicular lysosomes, thus meeting an even higher-than-normal need for autophagic flux.

Consistent with the model that TLs are activated in situations where high levels of autophagy are demanded, autophagic lysosomes also shift from vesicular to tubular form in several tissues, including the intestine, during starvation (Dolese et al., 2021; Villalobos et al., 2021 Preprint). In starved *C. elegans*, TLs labeled by Spin protein homologs receive and digest peroxisomes, and perhaps other cellular cargo, within hours of the starvation stimulus being introduced (Dolese et al., 2021). Unlike TLs associated with ALR, autophagic TLs induced by starvation in *C. elegans* are acidic and are induced by mTOR inhibition, rather than mTOR activation (Dolese et al., 2021; Villalobos et al., 2021 Preprint). Notably, some starvation regimens, including dietary restriction, trigger long-lasting health benefits. In several species, individuals subjected to dietary restriction often live longer than well-fed counterparts (Weindruch et al., 1986; Hansen et al., 2008; Madeo et al., 2015; Kapahi et al., 2017; Akagi et al., 2018). TLs appear crucial to this response; *C. elegans* genetic models of dietary restriction show constitutive TL induction in the intestine, and inhibiting TL formation prevents the full lifespan-extension effects of dietary restriction (Villalobos et al., 2021 Preprint). There is some evidence that starvation-dependent TL induction may carry transgenerational implications. When starved animals are re-fed and allowed to reproduce, autophagic TLs persist in a subset of well-fed descendants for several generations and can be used as a predictor of enhanced lifespan among progeny (Villalobos et al., 2021 Preprint). As with analysis of *Drosophila* VCP and SVIP, these observations hint that TLs execute a pro-health activity. Indeed, overexpressing *Drosophila* *SVIP* in *C. elegans* artificially stimulates TLs even in well-fed animals and improves aspects of late-age health (Villalobos et al., 2021 Preprint), suggesting autophagic TLs may be manipulated to prolong health during aging.

## Future Perspectives

The growing description and analysis of TLs in various species is beginning to alter how we view this essential organelle and its place within the landscape of a cell. Despite exciting advances, much is still unknown about the relationship between different types of TLs (Figure 1) and their possible connections to vesicular lysosomes. It is likely that some tubular forms may have a unique molecular make-up that allows them to assume this atypical morphology and to carry out distinct activities. For example, while traditional lysosome markers, including LAMP proteins, poorly label autophagic TLs despite localizing to TLs involved in ALR, other proteins, including Spin as well as some lysosomal hydrolases and membrane-fusion machinery, robustly mark degradative tubules, and may even play a stimulatory role in their induction (Rong et al., 2011; Johnson et al., 2015; Miao et al., 2020; Murakawa et al., 2020; Dolese et al., 2021). To date, only a few proteins are known to be essential for TL formation in the diverse cell types where they have been described (*see* Table 1). Identifying the full repertoire of genes required for TL assembly in different contexts may clarify unique elements of composition and/or regulation.

**TABLE 1:** Proteins that associate with lysosomes and directly modulate TLs in immune cells (iTLs), lysosome reformation (LR), and/or autophagy (aTLs).

	Protein name	Function	iTLs	LR	aTLs	Species	References
Cytoskeleton proteins	Kinesin-1	plus-end motor protein	✓	✓	?	mammals	(Hollenbeck and Swanson 1990; Du et al., 2016)
	Dynein	minus-end motor protein	✓	?	?	mammals	(Jordens et al., 2001; Mrakovic et al., 2012)
	Rab7	GTPase	✓	↓	✓	mammals, *Drosophila*	(Yu et al., 2010; Mrakovic et al., 2012); unpublished observation in *Drosophila*
	Arl8B	GTPase	✓	✓	?	mammals	Mrakovic *et al.* (2012)
	RILP	Rab7 effector	✓	?	?	mammals	Jordens *et al.* (2001)
	FYOC1	Rab7 effector	✓	?	?	mammals	Pankiv *et al.* (2010)
	ORP1L	Rab7 effector	✓	✓	?	mammals	Levin-Konigsberg *et al.* (2019)
	SKIP	Arl8B effector	✓	?	?	mammals	Mrakovic *et al.* (2012)
	ALG-2	EF-hand protein	✓	✓	?	mammals	Li *et al.* (2016)
	WHAMM	actin nucleation promoting factor	?	✓	?	mammals	Dai *et al.* (2019)
Lysosomal membrane proteins	LAMP-1	lysosome-associated membrane protein	✓	✓	?	mammals	(Vyas et al., 2007; Yu et al., 2010)
	SLC15A3	H^+^/oligopeptide symporter	✓	?	?	mammals	Nakamura *et al.* (2014)
	SLC15A4	H^+^/oligopeptide symporter	✓	?	?	mammals	Nakamura *et al.* (2014)
	Spinster	sugar transporter	?	✓	✓	mammals, *Drosophila,C. elegans*	(Rong et al., 2011; Murakawa et al., 2020; Dolese et al., 2021)
	TRPML	lysosomal Ca^2+^ channel	✓	✓	✓	mammals, *Drosophila, C. elegans*	(Li et al., 2016; Murakawa et al., 2020; Sun et al., 2020)
	V-ATPase	lysosomal H^+^ pump	?	?	✓	*Drosophila,C. elegans*	(Miao et al., 2020; Murakawa et al., 2020)
Lipid/sugar modifiers	PI3K	lipid kinase	?	↓	?	mammals	Munson *et al.* (2015)
	INPP5K	inositol phosphatase	?	✓	?	mammals	McGrath *et al.* (2021)
	PIP5K-1A and PIP5K-1B	lipid kinases	?	✓	?	mammals	Rong *et al.* (2012)
	PIP3Kβ	lipid kinase	?	✓	?	mammals	Sridhar *et al.* (2013)
Lysosomal membrane binding proteins	Clathrin	membrane binding	?	✓	✓	mammals,*Drosophila*	(Rong et al., 2012; Johnson et al., 2015)
	Syntaxin 17	SNARE	?	?	✓	*Drosophila*	Murakawa *et al.* (2020)
	BLOC1S1	biogenesis of lysosomal organelle complex-1	?	✓	?	mammals	Wu *et al.* (2021)
	SPG15	Spastizin	?	✓	?	mammals	Chang *et al.* (2014)
	SPG11	Spatacsin	?	✓	?	mammals	Chang *et al.* (2014)
	VCP	AAA+ ATPase	?	?	✓	*Drosophila*	Johnson *et al.* (2015)
	SVIP	VCP adapter	?	?	✓	*Drosophila*	Johnson *et al.* (2021)

(✓) indicates that the protein is required for lysosome tubulation in a particular context, (↓) indicates that downregulation of the protein is required for lysosome tubulation in a particular context, and (?) indicates that potential involvement of the protein has not been described or is unknown in a particular context.

Moving forward, further elucidating the purpose of this distinctive tubular organelle in different situations should provide fundamental information on cell biology and its larger relevance to animal physiology. Interestingly, TLs may not be present in all cell types; even in cells where they do form, their activity may be unique, and their induction may show differences in timing or scope. The observation that degradative TLs are stimulated in cases where there is high autophagic demand indicates that they may be preferential sites for bulk turnover of material. If so, it is unclear why TLs also appear stimulated by aging (Sun et al., 2020), when lysosome dysfunction abounds (Baxi et al., 2017). It is possible that TLs are deployed during aging in an attempt to rectify an increasing load of molecular stress and damage, but that their activity becomes saturated.

Finally, the development of strategies to induce TLs artificially, either genetically or pharmacologically, may lead to faster breakthroughs in TL biology. Overexpression of *SVIP* provides a genetic strategy to induce TLs and has revealed important roles for TLs in promoting healthy aging (Villalobos et al., 2021 Preprint). Excitingly, a DNA nano-device called *tudor* was developed that can artificially induce lysosome tubulation independent of any other biological stimulant and provides the first pharmacological strategy to induce TLs (Suresh et al., 2021). Mechanistically, *tudor* is internalized *via* receptor-mediated endocytosis and stimulates TLs *via* the PI3K–Akt–mTOR cascade, mirroring the macrophage TL induction mechanism (Suresh et al., 2021). The ability to induce TLs in the absence of inducing autophagic or inflammatory responses is a powerful tool to reveal core principles of TLs without triggering major cellular reprogramming. Indeed, this study revealed MMP9 as a missing link between macrophage activation and PI3K-Akt signaling (Suresh et al., 2021). Moreover, the use of *tudor* allowed visualization of inverse pH and Ca^2+^ spatial gradients along the tubules, a phenomenon that had been predicted but never observed directly (Suresh et al., 2021). The use of these new genetic and pharmacological technologies in different systems might not only reveal new principles underlying the function of TLs but could also be utilized as a tool to artificially elicit TL function in non-native contexts. Finding ways to boost TL performance pharmacologically could pave the way for therapeutic avenues to enhance cellular homeostasis and quality control, particularly in degenerative-disease states or in older individuals.
